# Strategies to prevent blood loss and reduce transfusion in emergency general surgery, WSES-AAST consensus paper

**DOI:** 10.1186/s13017-024-00554-7

**Published:** 2024-07-16

**Authors:** Federico Coccolini, Aryeh Shander, Marco Ceresoli, Ernest Moore, Brian Tian, Dario Parini, Massimo Sartelli, Boris Sakakushev, Krstina Doklestich, Fikri Abu-Zidan, Tal Horer, Vishal Shelat, Timothy Hardcastle, Elena Bignami, Andrew Kirkpatrick, Dieter Weber, Igor Kryvoruchko, Ari Leppaniemi, Edward Tan, Boris Kessel, Arda Isik, Camilla Cremonini, Francesco Forfori, Lorenzo Ghiadoni, Massimo Chiarugi, Chad Ball, Pablo Ottolino, Andreas Hecker, Diego Mariani, Ettore Melai, Manu Malbrain, Vanessa Agostini, Mauro Podda, Edoardo Picetti, Yoram Kluger, Sandro Rizoli, Andrey Litvin, Ron Maier, Solomon Gurmu Beka, Belinda De Simone, Miklosh Bala, Aleix Martinez Perez, Carlos Ordonez, Zenon Bodnaruk, Yunfeng Cui, Augusto Perez Calatayud, Nicola de Angelis, Francesco Amico, Emmanouil Pikoulis, Dimitris Damaskos, Raul Coimbra, Mircea Chirica, Walter L. Biffl, Fausto Catena

**Affiliations:** 1 General Emergency and Trauma Surgery Department, Pisa University Hospital, Via Paradisia, 56124 Pisa, Italy; 2 Anesthesiology and Critical Care, Rutgers University, Newark, NJ USA; 3 General Emergency and Trauma Surgery Department, Monza University Hospital, Monza, Italy; 4 Ernest E. Moore Shock Trauma Center, University of Colorado, Denver, CO USA; 5 General Emergency and Trauma Surgery Department, Cesena Hospital, Cesena, Italy; 6 General Surgery Department, Rovigo Hospital, Rovigo, Italy; 7 General Surgery Department, Macerata Hospital, Macerata, Italy; 8 General Surgery Department, University Hospital St George, Medical University, Plovdiv, Bulgaria; 9 Clinic of Emergency Surgery, University Clinical Center of Serbia, Belgrade, Serbia; 10 The Research Office, College of Medicine and Health Sciences, United Arab Emirates University, Al-Ain, United Arab Emirates; 11 Vascular and Trauma Surgery, Orebro Hospital, Orebro, Sweden; 12 Department of General Surgery, Tan Tock Seng Hospital, Singapore, Singapore; 13 Department of Trauma and Burns, Inkosi Albert Luthuli Central Hospital and Department of Surgical Sciences, University of KwaZulu-Natal, Durban, South Africa; 14 Anesthesia Department, Parma University Hospital, Parma, Italy; 15 General, Acute Care, Abdominal Wall Reconstruction, and Trauma Surgery Foothills Medical Centre, Calgary, AB Canada; 16 Department of General Surgery, Royal Perth Hospital, Perth, Australia; 17 Department of Surgery No. 2, Kharkiv National Medical University, Kharkiv, Ukraine; 18 General Surgery Department, Melahiti Hospital, Helsinki, Finland; 19 Emergency Surgery Department, Radboud Medical Centre, Nijmegen, The Netherlands; 20 Hillel Yaffe Medical Center, Rappaport Medical School, Haifa, Israel; 21 Division of General Surgery, School of Medicine, Istanbul Medeniyet University, Kadikoy, Istanbul, Turkey; 22 ICU Department, Pisa University Hospital, Pisa, Italy; 23 Emergency Medicine Department, Pisa University Hospital, Pisa, Italy; 24 Trauma and Acute Care Surgery, Foothills Medical Center, Calgary, AB Canada; 25 Unidad de Trauma y Urgencias, Hospital Dr. Sótero del Río, Santiago de Chile, Chile; 26 Department of General, Thoracic and Transplant Surgery, University Hospital of Giessen, Giessen, Germany; 27 General Surgery Department, Legnano Hospital, Legnano, Italy; 28 First Department of Anesthesiology and Intensive Therapy, Medical University of Lublin, Lublin, Poland; 29 Medicina Trasfusionale, IRCCS-Ospedale Policlinico San Martino, Genoa, Italy; 30 Department of Surgical Science, University of Cagliari, Cagliari, Italy; 31 Department of Anesthesia and Intensive Care, Parma University Hospital, Parma, Italy; 32 General, Emergency and Trauma Surgery Department, Rambam Medical Centre, Tel Aviv, Israel; 33 Trauma Surgery, Hamad General Hospital, Doha, Qatar; 34 Department of Surgical Diseases No. 3, University Clinic, Gomel State Medical University, Gomel, Belarus; 35 Harborview Medical Center, University of Washington, Seattle, WA USA; 36 Emergency Surgery, Ethiopian Air Force, Bishoftu, Ethiopia; 37 Department of Digestive and Emergency Surgery, Infermi Hospital, Rimini, Italy; 38 Trauma and Acute Care Surgery Unit Department of General Surgery, Hadassah Medical Center, Jerusalem, Israel; 39 Faculty of Health Sciences, Valencian International University (VIU), Valencia, Spain; 40 Division of Trauma and Acute Care Surgery, Department of Surgery, Fundación Valle del Lili, Cali, Colombia; 41 Hospital Information Services for Jehovah’s Witnesses, Tuxedo Park, NY USA; 42 Department of Surgery, Tianjin Nankai Hospital, Nankai Clinical School of Medicine, Tianjin Medical University, Tianjin, China; 43 Critical Care Division Hospital General de México Dr Eduardo Liceaga, Mexico City, Mexico; 44 General Surgery Department, Ferrara University Hospital, Ferrara, Italy; 45 Discipline of Surgery, The University of Newcastle, Newcastle, Australia; 46 3rd Department of Surgery, Attikon General Hospital, National and Kapodistrian University of Athens (NKUA), Athens, Greece; 47 General Surgery Department, Edinburgh Hospital, Edinburgh, UK; 48 General Surgery Department, Riverside University Health System Medical Center, Loma Linda, CA USA; 49 General Surgery Department, Grenoble University Hospital, Grenoble, France; 50 Division of Trauma/Acute Care Surgery, Scripps Clinic Medical Group, La Jolla, CA USA

**Keywords:** Blood management, Mortality, Morbidity, Policy, Management, Jehovah’s witnesses, Religion, Refusal, Optimization

## Abstract

Emergency general surgeons often provide care to severely ill patients requiring surgical interventions and intensive support. One of the primary drivers of morbidity and mortality is perioperative bleeding. In general, when addressing life threatening haemorrhage, blood transfusion can become an essential part of overall resuscitation. However, under all circumstances, indications for blood transfusion must be accurately evaluated. When patients decline blood transfusions, regardless of the reason, surgeons should aim to provide optimal care and respect and accommodate each patient’s values and target the best outcome possible given the patient’s desires and his/her clinical condition. The aim of this position paper was to perform a review of the existing literature and to provide comprehensive recommendations on organizational, surgical, anaesthetic, and haemostatic strategies that can be used to provide optimal peri-operative blood management, reduce, or avoid blood transfusions and ultimately improve patient outcomes.

## Introduction

Globally, it is estimated that there are 313 million surgical procedures performed annually. Of which, an estimated 7.7% of these case [4.2 million people] will die within the first 30 days after surgery [1, 2]. Whilst there is no definitive consensus data on postoperative morbidity, this is expected to be much higher [3, 4].

Emergency general surgeons often provide care to severely ill patients requiring surgical interventions and of intensive physiological support. While various patients and operative factors are at play in emergency general surgery, one of the primary drivers of morbidity and mortality is perioperative bleeding. This can manifest preoperatively in hemodynamically unstable trauma patients for example, or intraoperatively in complex emergency surgical procedures or post-operatively in any surgical patient. Bleeding contributes to significant morbidity, mortality and consumption of resources [5–8]. Very often in supporting physiology of the patients’ blood transfusions are applied liberally. Although helpful in some scenarios, large volume blood transfusions can also have detrimental effects. Transfusion can have a negative impact on clinical outcomes, economics, and on resource allocation. Moreover, considering the importance of the physician–patient relationship, it is necessary to respect the patient’s will and concerns and to understand and appropriately address any special patient’s needs that arise. Patient autonomy must be completely respected by all those providing healthcare while providing high-quality, effective, and safe treatment. In general, when addressing life threatening haemorrhage, blood transfusion can become an essential part of overall resuscitation. However, under all circumstances, indications for blood transfusion must be accurately evaluated. When patients decline blood transfusions, regardless of the reason, surgeons should aim to provide optimal care and respect and accommodate each patient’s values and target the best outcome possible given the patient’s desires and his/her clinical condition [9]. All those points should be accurately discussed and agreed with the patient in an honest and ethical manner. In elective surgery, there are now many evidence that support the implementation of strategies to reduce use of blood in peri-operative period. The 3 pillars of Patient Blood Management (PBM) was proposed for pre-, intra- and post-operative period: optimize erythropoiesis, minimize blood loss and bleeding, optimize physiological tolerance to anemia [10]. In an emergency setting, only a part of these recommendations can be maintained, because pre-operative period is very short and there is no time to prepare patient (Table 1).

**Table 1:** Blood sparing strategies in emergency setting

Preoperative	Detect and manage anemia
	Identify and manage underlying bleeding diathesis and bleeding risk
	Refer for further evaluation if necessary
	Apply Massive transfusion protocols
	Apply risk scores
Intraoperative	Meticulous hemostasis and surgical techniques
	Blood-sparing surgical devices
	Anesthetic blood-conserving strategies
	Autologous blood options
	Maintain normothermia
	Pharmacological/hemostatic agents
	Viscoelastic test
	Optimize cardiac output
	Optimize ventilation and oxygenation
	Optimize patient position
Postoperative	Optimize erythropoiesis
	Be aware of drug interactions that can increase anemia
	Vigilant monitoring and management of postoperative bleeding
	Avoid secondary hemorrhage
	Rapid warming, maintain normothermia (unless hypothermia specifically indicated)
	Minimize iatrogenic blood loss
	Hemostasis/anticoagulation management
	Prophylaxis of upper GI hemorrhage
	Avoid/treat infections promptly
	Optimize anemia reserve
	Maximize oxygen delivery
	Minimize oxygen consumption
	Restrictive transfusion thresholds

Notes on the use of the position paper

The aim of this position paper was to perform a review of the existing literature and to provide comprehensive recommendations on organizational, surgical, anaesthetic, and haemostatic strategies that can be used to provide optimal peri-operative blood management, reduce, or avoid blood transfusions and ultimately improve patient outcomes.

The position paper presents the methods for optimal management of blood products. It doesn’t represent a standard of practice. It is a suggested plan of care, based on best available evidence and the consensus of experts, but they do not exclude other approaches as being within the standard of practice. For example, they should not be used to compel adherence to a given method of medical management but assist in determining which method should be finally determined after taking account of the conditions at the relevant medical institution (staff levels, experience, equipment, etc.) and the characteristics of the individual patient. However, responsibility for the results of treatment rests with those who are directly engaged therein, and not with the consensus group.

## Methods

A computerized search was done by a bibliographer in different databases (MEDLINE, Scopus, EMBASE) and citations published between January 2000 to May 2023 were included when satisfying, the primary search strategy: ‘transfusion’, ‘blood management’, ‘blood conservation’, ‘blood loss’, ‘haemoglobin’, ‘hemoglobin’, “guidelines”, combined with AND/OR. No search restrictions were imposed. Expert opinion reviews, narrative reviews, case reports and case series based on less than 30 patients were not considered relevant. The dates were selected to allow comprehensive published abstracts of clinical trials, consensus conference, comparative studies, congresses, guidelines, government publication, multicenter studies, systematic reviews, meta-analysis, large case series, original articles, and randomized controlled trials (RCT). Narrative review articles were only used to determine if other cited studies should be included.

The quality of evidence and the recommendations provided were evaluated according to the GRADE methodology [11]. A group of experts in the field led by a central coordinator was contacted to express their evidence-based opinion. Through the Delphi process, different issues were discussed in subsequent rounds. The central coordinator assembled the different answers derived from each round. Each version was then revised and improved. The final version about which the agreement was reached resulted in the present manuscript.

### Recommendations

There are many potential areas of improvement and intervention in this topic. We hereby present the recommendations systematically by grouping them broadly into surgical and anaesthetic strategies in all perioperative phases.

### Preoperative interventions

#### Recommendation 1: perform a detailed pre-operative assessment with particular focus on identifying patients who might have underlying bleeding diathesis. (Grade of recommendation: 1B)

The following specific information should be obtained from the patient and or the relatives during the clinical history review [12, 13]: (I) History of previous blood transfusion or bleeding events [eg easy bruising, easy gum bleeding]; (II) History of medications associated with increased risk of bleeding (*e.g.*, warfarin, direct oral anticoagulant (DOAC), clopidogrel, aspirin and other antiplatelet or platelet inhibitors, as well as vitamins or herbal supplements that may affect coagulation; (III) Presence of congenital coagulopathy; (IV) History of thrombotic events (*e.g.,* deep vein thrombosis, pulmonary embolism); (V) Risk factors for organ ischemia (*e.g.*, cardiorespiratory disease) which may influence the ultimate transfusion threshold for red blood cells (*e.g.*, haemoglobin level). A direct physical examination of the patient is essential.

To evaluate the presence of ecchymosis, petechiae, pallor. Pre-operative laboratory tests are strongly suggested to evaluate haemoglobin, haematocrit, and coagulation screening. Anaemia work-up panels should be included whenever appropriate (Refer to Recommendation 3). For all the patients with any suspicious history or findings a more detailed description of symptoms should be obtained. If issues are confirmed or remain suspected referral to an expert in coagulation disorders is suggested.

#### Recommendation 2: apply risk scores whenever appropriate to identify high risk patients. (Grade of recommendation: 1C)

To complement the anamnesis and to ensure a more comprehensive identification of patients with potential bleeding issues bleeding assessment scores are suggested. The International Society on Thrombosis and Haemostasis Bleeding Assessment Tool [14], the Vicenza Bleeding Score [15] and the Paediatric Bleeding Questionnaire [16] are well-known and generally considered to be reliable and valid tools for assessing bleeding risk. Although none of these have been validated prospectively in a large population of general surgical patients, the suggested specific questions contained within them are likely to be superior to an unstructured bleeding evaluation and scoring [17]. Other well-known examples of validated tools exist as the Papworth Bleeding Risk Score and the ACTA-PORT score [18, 19]. Unfortunately, their use has been restricted to cardiac surgery. Well validated bleeding risk scores in the field of emergency general surgery are strongly needed.

#### Recommendation 3: institute proactive preoperative interventions to pre-emptively minimize blood loss and to reduce potential transfusion (Grade of recommendation: 1A).

Several proactive actions must be undertaken as soon as possible by physicians and surgeons to optimize patient care and prevent blood loss.

##### Identification and treatment of anaemia

Anaemia is defined as per the World Health Organization (WHO) using the following haemoglobin thresholds [20, 21]:

- 11.0 g/dl for children 0 – ≤ 5 years old.
- 11.5 g/dl for children 5 – ≤ 12 years old.
- 12.0 g/dl for children 12 – ≤ 15 years old, and non-pregnant women ≥ 15 years old.
- 11.0 g/dl for pregnant women.
- 13.0 g/dl for men ≥ 15.0 years old.

Anemia is a common feature in patients undergoing non cardiac surgery with a prevalence of 27–30%. Even if mild anemia, has been associated with an increased risk of allogeneic red blood cell transfusion and increased risk of mortality and morbidity after surgery. Anemia can be classified into four categories: (1) iron deficiency anemia; (2) anemia of chronic disease; (3) anemia of chronic disease with iron deficiency; (4) anemia of other causes. Preoperative screening for iron deficiency is reccomended. Anaemia screening blood tests should be done to identify the types of anaemia, and thereafter to plan the adequate treatment. Blood transfusion is generally considered the fastest way to treat anaemia. However, several concerns exist about this topic; types of transfusion and other available therapeutic modalities will be explored in subsequent recommendations. Selective administration of erythropoietin and/or iron to improve preoperative haemoglobin levels can be useful. Several studies including meta-analyses of placebo-controlled randomized controlled trials (RCT) indicate that erythropoietin with or without iron is effective in reducing the number of patients requiring allogeneic transfusions as well as reducing the volume of allogeneic blood transfused [22–36]. Conversely, some RCTs reported equivocal findings when preadmission oral iron is compared with either placebo or no iron regarding preoperative haemoglobin levels or perioperative allogeneic blood transfused [37–39]. In certain emergency surgical situations, there may not be sufficient time to administer some treatments and transfusion may remain the best and only solution in those patients accepting blood products administration. Whenever possible a multidisciplinary, multimodal, programmatic approach to perioperative anemia should be considered.

##### Discontinuation of anticoagulants and antiplatelet agents

In general, whenever possible discontinuation of all antiplatelets (i.e. aspirin, clopidogrel) and anticoagulants (i.e. warfarin, direct oral anticoagulants (DOACs)) in the perioperative phase is the best option. The decision and timing of interruption are influenced by patient’s characteristics, types of surgery, type of anaesthesia (i.e. general vs regional) and type of anticoagulant., some patients direly need these drugs to be continued to reduce the risk of major cardiovascular and thrombotic events. In these cases, the decision needs to be tailored and multidisciplinary and tailored to the patient’s specific condition. The intrinsic risks of suspending these agents must be balanced against the procedure-related risks of bleeding. The discussion about the risk–benefit balance must be done together with the patient and appropriate specialists. In general, due to its pharmacokinetics aspirin can be continued for most of the emergency surgical procedures. Its discontinuation may be considered in the post-operative period according to the risk–benefit balance. Regarding warfarin, stopping it and bridging should be considered only in patients with the highest risk of thrombosis (i.e. those with mechanical heart valves or venous thromboembolism within the previous 3 months); postoperative bridging should not be started until at least 48 h after surgery with a high bleeding risk.

DOACs, owing to their predictable pharmacokinetics, should be stopped 48 h before most operations in the presence of normal hepatic and renal function. Adenosine 5^′^-diphosphate receptor antagonists, such as clopidogrel, should be stopped 5–7 days before operation. In emergency surgery, there is not enough time to stop anticoagulant drugs and the risk of bleeding is increased. In this situation, specific reversal factors are available and the decision to reverse must considerthe balance of bleeding versus the risk of clotting.

##### Reversal of anticoagulants

Reversal of anticoagulants includes several options to be deployed individually or in combination: a) administration of prothrombin complex concentrates [PCCs]; b) administration of fresh frozen plasma [FFP]; c) administration of vitamin K; d) administration of tranexamic acid; e) specific anticoagulants reversal factors. No definitive data exist about the different potential advantages and drawbacks of these anticoagulants’ reversal therapies in emergency setting. Observational studies and case reports indicated that four-factor PCCs administered pre-operatively will reduce International Normalized Ratio (INR) values due to warfarin, with thromboembolic events reported in 0.003% of patients [40–42]. No sufficient data exist to evaluate the impact of the use of FFP with reversal of anticoagulants in emergency setting. In general, it may take up to 90 min for the ABO blood group type to match and thaw plasma. From the time of the transfusion order to the administration. Adequate plasma for VKA reversal is 15 to 30 mL/kg; this dose is not practical for rapid VKA reversal, so a plasma concentration of 10 to 15 mL/kg is used more commonly. However, despite the volume reduction, potential adverse effects of plasma transfusion include circulatory volume overload, allergic reactions, and risk of transfusion-related acute lung injury. Because these effects are not observed with PCC, it is used preferentially, particularly in volume-sensitive patients [43]. One retrospective study comparing vitamin K administered immediately before surgery with no vitamin K administered reports equivocal findings for transfusion requirements and is therefore more suited for non-urgent cases [44]. Conversely, tranexamic acid should be considered in patients undergoing urgent surgery with a high risk of bleeding who are on antiplatelet agents or where a residual anticoagulant effect of DOACs is suspected [45, 46]. In the UK, tranexamic acid is recommended for all surgery where blood loss is expected to be greater than 500 ml [47, 48]. However, TXA may be associated with thrombotic events. Hemoadsorption techniques like CytoSorb can rapidly reverse anticoagulation in emergency surgeries by filtering blood-borne substances, including anticoagulants, thus mitigating bleeding risks. Overall, the decision to reverse anticoagulants must be defined on a case-by-case basis.

##### Antifibrinolytics for prophylaxis of excessive blood loss

###### Tranexamic acid

In emergency surgery settings, meta-analysis and RCTs indicate that tranexamic (TXA) acid for prophylaxis of excessive bleeding administered before and/or during a procedure is effective in reducing perioperative blood loss by approximately one-third [49], the number of patients transfused, and the volume of transfused blood products. Most RCTs comparing TXA with placebo or no TXA controls report no differences for stroke, myocardial infarction, renal failure, reoperation for bleeding, or mortality. However, there are RCT studies reporting increased thrombotic risk and observational studies suggesting adverse outcomes [50–53].

What remains unclear is the optimal route, dose, and timing of TXA administration. Protocols in studies to date are extremely heterogeneous and its administration can be via intravenous, intra-articular, oral routes or in combination. Doses may also be weight-adjusted and can also range from 10 to 20 mg/kg. Administration can also be repeated during the intraoperative and postoperative phases [48]. Some studies even suggest topical TXA delivery to overcome contraindications such as renal disease owing to lower plasma levels [26].

###### ε-Aminocaproic acid [and Aprotinin]

Meta-analysis of placebo-controlled RCTs indicate that the use of ε-aminocaproic acid, which similar to TXA inhibits plasminogen binding to fibrin, administered before and/or during a procedure is effective in reducing total perioperative blood loss and the number of patients transfused in major elective cardiac, orthopaedic, or liver surgery; equivocal findings are reported for the volume of blood transfused [54–63]. No data exist about its use in emergency surgery settings. However, some data suggest that Aprotinin, which inhibits plasmin directly, may be associated with increased postoperative mortality rates [64].

###### Desmopressin (DDAVP; 1‐deamino‐8‐D‐arginine‐vasopressin)

Desmopressin (also known as DDAVP or 1-deamino8-D-arginine vasopressin) has been used for the treatment of congenital bleeding disorders for almost four decades. It acts by increasing plasma von Willebrand factor, factor VIII and intracellular platelet calcium/ sodium ion concentrations, and by increasing formation of procoagulant platelets and platelet adhesion to collagen under flow [65] A recent Cochrane review comparing DDAVP versus placebo or no treatment for people with platelet dysfunction showed that DDAVP may lead to a reduction in the total volume of red cells transfused (low‐quality evidence) and in total blood loss (low‐quality evidence) [66]. This effect is reduced when DDAVP is given to patients without platelets dysfunction, indeed It may slightly decrease the total volume of red cells transfused in adult cardiac surgery but may lead to little or no difference in orthopedic surgery, vascular surgery, or hepatic surgery. In conclusion it is possible that people who are more vulnerable to bleeding, such as those taking antiplatelet agents, may gain more benefit from DDAVP [66]. A recent review supports desmopressin as potentially beneficial with minimal risk for use in patients with antiplatelet-associated intracranial hemorrhage.

It can be given subcutaneously or intravenously at a dose of 0⋅3 μg/kg. Safety considerations include the risk of developing arterial or venous thrombosis and, in rare cases, desmopressin may be associated with hyponatremia and seizures. Recently, its use has been expanded to other potential indications. In trauma, European guidelines [67] recommend administration of desmopressin to patients on antiplatelet agents. In the perioperative setting, guidelines [13] suggest using desmopressin where there is demonstrable evidence of acquired platelet dysfunction secondary to drugs, uraemia, or cardiopulmonary bypass. However, the evidence for the ability of desmopressin to reduce perioperative transfusion requirements and blood loss is weak especially in emergency settings; moreover, the indications at present are to administer only one dose and only in selected cases [65, 66].

(e) Opportunity for preadmission autologous blood donation

RCTs have shown that the preadmission donation of autologous blood reduces the number of patients requiring allogeneic transfusions and the volume of allogeneic blood transfused per patient [68–71]. However, its use in emergency general surgery is generally unapplicable, although the concept of the walking blood bank, used widely in the military, is now being discussed for civilian application.

(f) Damage control resuscitation
  Damage control resuscitation is a strategy that should be applied both in the prehospital and in the in-hospital setting [72, 73]. It aims at first to increase survival by reducing the physio-metabolic impact of extensive bleedings and as a positive collateral outcome it may reduce the total amount of blood products transfused. In fact, it is demonstrated that the decrease in blood products usage is associated to reduced mortality [74]. At present the cornerstones of this strategy focus on temporary hemorrhage-control devices and early use of whole blood or balanced blood product-based transfusions. This brings a reduction in crystalloid use through also the application of hypotensive resuscitation protocols aiming to promote hemostasis and decrease coagulopathy. All these procedures in general help in correcting physio-metabolic impairment and allow a rapid and definitive hemorrhage control. Damage control resuscitation is part of to the more general damage control management strategy that must be structurally applied and must be multidisciplinary; in order to obtain the best results, it should be participated by all the actors of the emergency and trauma patient management [75–77].
  However, for many years, a discussion on the benefit of ratios for damage control resuscitation has become the center of attention. Controversies about the true benefits of this approach have been addressed in several research. Since the publication of The Pragmatic, Randomized Optimal Platelet and Plasma Ratios trial, a balance of 1:1:1 ratio of red blood cells, plasma, and platelets has become the standard of care despite a lack of differences in the trial’s primary outcome.Like this study, other retrospective data have supported these findings over the last decade, adding to the body of evidence on the importance of balancing blood product administration for acutely bleeding trauma patients. The PROPPR study only showed a benefit on survival at 6 h with no benefit in the first analysis for a 30-day survival rate; a secondary analysis found a possible benefit in the 30-day survival rate [78].
  Several studies have found a dose-dependent relationship between blood transfusion and mortality. Schneider et al. found that the mortality was significantly higher in the Massive Blood Transfusion (MBT defined as patients that received at least 10 PRBC in the first 4 h of admission group (39.4% versus 14.5%; P < 0.001), and for each additional PRBC unit transfused in the OR increased by 1.08 (95% confidence interval 1.08–1.09, P < 0.001) for mortality. In patients receiving 6 to 10 units of red blood cells, red blood cell: platelet ratios were not associated with 4-h mortality, and only red blood cell:fresh frozen plasma ≥ 4:1 were associated with significantly higher odds of 4-h mortality compared to 1:1. For patients receiving > 10 red blood cell units, increasing red blood cell:platelet and red blood cell:fresh frozen plasma ratios were consistently associated with increased odds of 4-h mortality. For example, in red blood cell volumes of 11 to 15, 16 to 20, and > 20 units, risk-adjusted 4-h mortality odds ratios for red blood cell:platelet ≥ 4:1 were 2.27 (1.47–3.51), 3.32 (2.26–4.90), and 3.01 (2.33–3.88), respectively [79]. For this reason resuscitation measures should be continued using a goal-directed strategy, guided by standard laboratory coagulation values and/or VEM [80]. For non-trauma related bleeding In the early treatment phase of uncontrolled massive elective surgery bleeding, they suggest massive transfusion [6 to 10 units] with a high ratio (1: 1) of plasma to RBCs; however, it is also recommended switching to a goal-directed transfusion strategy (based on Hb and/or physiological RBC transfusion triggers, coagulation factor substitution and platelet transfusion triggers) as soon as possible, to reduce risk of massive transfusion.

#### Recommendation 4: institute hospital-wide blood management protocols at the organizational level to optimise transfusion and blood product utilization. (Grade of recommendation: 1A)

It is imperative that hospitals have clear cut institutionalized multi-modal protocols, defined as strategies that typically consist of a predetermined “bundle” of interventions, that all members should refer and adhere to. This may potentially improve a collective effort towards effective blood management. These protocols should be implemented for preventing or reducing blood product inappropriate usage and transfusion requirements. These bundles should include multidisciplinary approach, institutional support, transfusion algorithms, and point-of-care testing. Visco-Elastic-Testing (VET) as Rotational thromboelastometry (ROTEM) or Thromboelastography (TEG)—guided protocols showed to reduced allogeneic blood product requirements [81–86]**.** Restrictive versus liberal transfusion strategies showed different definitions but. In general haemoglobin criteria for transfusion between 7–8 g/dl [87] and haematocrit values less than 25% are typically reported as restrictive. Specific criteria must be applied to some subgroups of patients. Meta-analysis of RCTs comparing restrictive with liberal transfusion criteria report fewer red blood cell transfusions when restrictive transfusion strategies are employed with no major drawbacks observed [88–92]. Protocols optimizing the blood product usage may also bring as a potential positive consequence that blood may be made available whenever really needed. In emergency surgical conditions is the case for Massive Transfusion Protocols (MTP) that have been implemented in cases of life-threatening haemorrhage mainly after trauma. They are intended to minimize the adverse effects of hypovolemia and dilutional coagulopathy [93]. Lastly, to further help in optimizing the blood product usage, the different institutions can determine a maximal surgical blood order schedule which has been shown to improve the efficiency of blood ordering practices [13, 44, 65–67, 94–98].

## Intraoperative surgical factors

### Recommendation 5: precise and meticulous surgical technique including the use of surgical adjuncts, may help in minimising surgical bleeding. (Grade of recommendation: 1C)

Accurate surgical technique and intimate knowledge of anatomy are fundamental in reducing blood loss. If they are combined with the wielding of advanced surgical devices and effective hemostatic materials blood loss can be kept to a minimum. Some of these tools will be mentioned here.

- Diathermy
  Monopolar and bipolar radiofrequency, gas-mediated or other electrosurgical tools are key for achieving haemostasis during surgery. There is however limited evidence that they alone may reduce blood loss and factors such as surgical technique are likely to be more important [70].
- Topical agents
  Topical agents including fibrin sealants (fibrinogen and thrombin), gelatine–thrombin matrices and oxidized cellulose may be applied to bleeding tissues during surgery as a hemostatic strategy. However, these agents are expensive and for this reason not everywhere available. Despite numerous studies across different surgical specialties, there is only weak evidence to suggest that they offer a clinically important reduction in blood loss especially in emergency surgery settings [71, 81]. Accurate technique and hemostasis remain the cornerstone.
- Use of laparoscopic and robotic surgery platforms
  Mini-invasive approaches have been developed and brought several positive outcomes. However, they seem of limited help in substantially reduce blood loss especially in emergency surgery [68, 69, 99]. To increase at best the bleeding during mini-invasive approaches an accurate and anatomical surgical technique is needed.
- Cell salvage
  Cell salvage is a method of recovering blood from the surgical field during the intraoperative or immediate postoperative phase that is then reinfused to the patient. Several indications and guidelines [47] recommend the use of cell salvage for procedures when a very high volume of blood loss is anticipated. Recent indications consider high volume a quantity of blood loss greater than 500 ml [28, 29]. The key principle is to use cell salvage in combination with other blood conservation strategies in trying to prevent or at least to reduce the likelihood of allogeneic blood transfusion and/or severe postoperative anemia [29]. Cell salvage reduces the risk of exposure to allogeneic blood by 54% across all surgical specialties including emergency surgery [30, 31].
  Emergency surgical procedures with significant variation in volume of blood loss make difficult to anticipate whether sufficient blood will be collected to permit reinfusion. In this setting, cell salvage can be used in collect-only mode and eventually process it for reinfusion only if in presence of more than 500 ml. There remain controversies regarding which patients benefit from cell salvage, and it is not currently recommended for routine use during all emergency surgical procedures [29, 32, 33]. Moreover, in those situations in facing potential infectious contamination or in high risk of cancer cells spread blood collection and reinfusion is not universally feasible. In fact, infection and malignancy were traditionally contraindications to blood cell salvage, but there is increasing evidence to support its use in these settings. With the use of a leucocyte depletion filter (40 μm), there is a 99% reduction in bacterial contamination in blood resuspended in 0⋅9% saline [34]. The potentially increased risks of bacterial contamination must be weighed against the increased risk of infection through immunomodulation secondary to allogeneic blood transfusion [35].
- Tourniquet
  Tourniquets are used widely during limb surgery, and emergency surgeons may need to use these devices in emergency and trauma situations. However, although tourniquets reduce intraoperative blood loss, meta-analyses of studies suggest that there is no difference in overall blood loss [100, 101]. The release of inflammatory mediators because of limb ischaemia may paradoxically increase blood loss [102]. Interpretation of studies is often limited by the heterogenous data regarding tourniquet application techniques in terms of inflating timing and pressures. Currently, the decision to use a tourniquet is dictated by several factors associated to the blood management, such as visibility of the surgical field. Disadvantages of tourniquet use include increased postoperative pain, impaired muscles function and increased risk of thrombotic events [100, 103, 104].
- Drains
  Surgical-site drains are often used with the aim of reducing hematomas and hypothetically reduce surgical-site infection that may potentially increase post-operative bleeding risks. A limited number of robust studies have investigated the effectiveness of drains in preventing postoperative complications, and many did not demonstrate any benefit of drain insertion over no drain [38, 39, 105, 106]. Conversely, drains may increase infection rates. Some studies have investigated the possibility to deploy autologous reinfusion drains for post-operative cell salvage [107, 108].
- Damage control surgery
  Damage control surgery (DCS) as part of damage control strategy is generally performed together with damage control resuscitation in severely bleeding patients [109–112].
  DCS should be implemented as soon as its necessity is recognized [113–116]. It basically reduces the surgical time and procedures only to those fundamental maneuvers aiming to control bleeding and potential contamination [117]. The patients are then transferred to intensive care unit to allow them to recover from the physiological derangements and to be able to face further reconstructive surgical phases. It is demonstrated that DCS helps in increasing overall survival of patients, reduces the risk of developing trauma coagulopathy and can potentially reduce the total amount of blood product usage [109, 110]. DCS strategies may be also applied to non-traumatic emergency surgical patients. Lastly it may fit also to those patients experiencing unexpected severe bleeding during other surgical procedure not amenable of definitive care due to the suddenly deteriorated physio-metabolic conditions.
- Endovascular control of bleeding
  Advances in interventional radiology techniques, employed by vascular and trauma surgeons as well, offer hyper-selective alternatives to surgery [118–120]. In fact, much of the bleeding once considered for open surgical control at present can be safely and definitively stopped with endovascular management [121–123]. This strategy, impact less on the physiology of the patient and allow for less invasive approaches. This in definitive reduces the relative and absolute bleeding. Moreover, endovascular approaches can be also applied to diagnose and prevent bleedings. In those situations where the bleeding is suspected but no source is evident the early application of angiography may help in diagnosis and subsequent treatment [124–128]. Lastly, early radiographic evaluation and eventual prophylactic angioembolization should be considered for injuries that may not currently be bleeding but with potential for deterioration or delayed hemorrhage [129–132]. Access to endovascular approaches should be developed and readily available for all complex procedures with risk of major bleeding.

## Intraoperative anaesthetic factors

### Recommendation 6: maintaining permissive hypotension can help minimise bleeding in the absence of moderate-severe brain trauma. (Grade of recommendation: 1C)

Permissive hypotension means maintaining a mean arterial blood pressure between 50 to 65 mmHg which can help reduce blood loss in severe trauma and during surgical procedures. Permissive hypotension, however, must be balanced against the risks of organ hypoperfusion [82]. This technique should be avoided in patients with moderate or severe brain trauma, coronary artery disease or cerebrovascular disease.

Permissive hypotension can be achieved through a reduction in cardiac output, blood pressure or a combination of these. Techniques include patient positioning, central neuraxial anesthesia, intravenous anesthetics (such as propofol), opioids (remifentanil), directly acting vasodilators (nitroglycerine), selective beta-blockers (esmolol), selective α-blocker (dexmedetomidine), combined α- and beta-blocker (labetalol) and volatile anesthetics (sevoflurane). Systematic reviews and RCTs have demonstrated that permissive hypotension reduces blood loss in patients undergoing orthopedic, maxillofacial, spinal, and urologic surgery [83–85]. Monitoring for perfusion of vital organs during emergency surgery help to minimize adverse effect of permissive hypotension. It includes standard ASA monitoring but also echocardiography, renal monitoring (urine output), cerebral monitoring (i.e., cerebral oximetry and near infrared spectroscopy [NIRS]), analysis of arterial blood gasses, and mixed venous oxygen saturation.

However, all the included studies were small, of low quality and, more importantly, did not directly evaluate the emergency surgical setting where unexpected sudden major losses in blood may occur.

## Recommendation 7: central neuraxial anaesthesia to help minimise bleeding. (Grade of recommendation: 1C)

Anaesthetic techniques play an important role in minimizing intraoperative blood loss. Central neuraxial blockade (subarachnoid/epidural anaesthesia) results in blockade of preganglionic sympathetic nerve fibres, arterial hypotension and reduced peripheral venous pressures. These result in less arterial and, perhaps more noticeably, less venous oozing from the surgical site. Blockade of sympathetic nerve fibres also results in attenuation of the surgical stress response, which in turn is associated with stabilization of clotting factors and reduced fibrinolysis [86, 88].A few meta-analyses [89, 90] of RCTs, conducted in several surgical specialties (abdominal, thoracic, pelvic, lower extremity), demonstrated that the use of neuraxial anesthesia was associated with significant decreases in intraoperative blood loss and allogeneic red blood cell transfusion requirements. This may not however, be feasible in all emergency surgical procedures. However, dedicated protocols may be applied to implement the usage of neuraxial anesthesia in selected cases.

## Recommendation 8: in the operating room, patient positioning is important to reduce bleeding; comorbidities and the eventual associated lesions must be taken into consideration. (Grade of recommendation: 1C)

Correct patient positioning is a simple and effective intervention to reduce intraoperative blood loss. Incorrect positioning may potentially even increase bleeding [133]. Changes of intra-abdominal pressure may also be associated with blood loss [134]. In addition, where possible, the site of surgery should be elevated above the level of the right atrium to facilitate venous drainage and reduce venous pressures; this is specifically true for orthopedic surgery. Very important to keep in mind are the patient eventual comorbidities and the potential associated lesions. In the case of brain trauma superelevation of the head over the body plane (of 20–30 degrees) may help in maintaining the best balance between the position and the associated lesions. For what concern abdominal lesions, no specific position exist that may increase the hemostasis or reduce the blood loss.

## Recommendation 9: it is important to avoid hypothermia to help minimise bleeding. (Grade of recommendation: 1B).

Intraoperative hypothermia is defined as a core body temperature below 36^∘^C and can result from many factors such as low operating theatre temperatures, evaporation from body cavities, use of cold intravenous fluids and anesthetic gases, reduced metabolic activity, and loss of thermal regulation and responses owing to anesthesia. Patients at risk of developing hypothermia include those at extremes of age, undergoing combined regional and general anesthesia, major surgery, prolonged surgery and with higher ASA fitness grade or emergency and trauma surgical patients. The reversible adverse effects of hypothermia on platelet function and the coagulation cascade, as a result of impairment of temperature-dependent enzymatic reactions, are well recognized [135, 136]. Even mild hypothermia has been associated with an increase in blood loss of 16 per cent, and increase in relative risk of red blood cell transfusion by 22 per cent [137, 138]. In addition, hypothermia can also lead to increased rates of wound infection [139] and cardiovascular events [140], and prolonged recovery [93]. Several indications exist about the potential actions to prevent intraoperative hypothermia and its consequences. NICE has issued guidance on the prevention and management of hypothermia in patients undergoing surgery. Examples of strategies used to avoid intraoperative cooling include regular temperature monitoring every 30 min, ensuring the ambient theatre temperature of at least 21^∘^C, using active forced air warming devices and administering intra- venous fluids through a fluid warmer.

## Recommendation 10: ventilation strategies are important to minimise bleeding. (Grade of recommendation: 1C)

Positive pressure ventilation with minimal use of positive end-expiratory pressure (PEEP) and low tidal volumes has been advocated to reduce blood loss as it theoretically promotes venous return [95]. However, this needs to be balanced against the benefits of PEEP, such as alveolar recruitment. A recent secondary analysis, of a previously published trial [96] comparing the effect of a lung-protective strategy in patients undergoing major abdominal surgery, evaluated the effect of PEEP between 6 and 8 cmH_2_O *versus* zero PEEP in patients undergoing hepatic resection surgery [97]. The authors found that using PEEP, compared with a zero PEEP strategy, was not associated with increased bleeding. These data however may not be directly translated to the emergency setting due to the intrinsic differences of the patient’s condition and the potential unfeasibility of such ventilation strategy. Tailored patient evaluation is mandatory and strict multidisciplinary collaboration is fundamental.

## Recommendation 11: procoagulant factors are useful to help minimise bleeding. (Grade of recommendation: 1C)

There is growing interest in the targeted use of procoagulant hemostatic factors, mainly driven by the demonstrable lack of efficacy and safety concerns regarding the use of fresh frozen plasma alone [40, 44]. These can be derived from plasma as concentrates or developed as specific recombinant factors. The potential advantages include more rapid availability in the emergency situations, as the current processes for thawing plasma do not apply, and better efficacy as a more concentrated source of factors is being administered. This would, in turn, lead to quicker and stronger fibrin clot formation. During active bleeding or in situations where physio metabolic derangements are present the clinical significance of the routine parameters PT, aPTT and platelet count is rather weak. Targeted use of coagulation factors or procoagulant products driven by thromboelastography or thromboelastometry evaluation is showing very good results in all the field of surgery either elective or emergency and trauma ones. Even in presence of strong evidence about the use of targeted infusion therapies, in the clinical everyday routine, a "blind" therapy is often applied. Consequently, a series of medications and blood products are administered consecutively until the bleeding stops. If the cause of the bleeding is not within the ones solvable by the administered products, it may result in useless medication and blood products. Therefore, the patient is potentially exposed to harmful preparations and costs may be unnecessarily increased.

Concentrates can contain multiple factors; for example, prothrombin complex concentrates contain three or four vitamin K-dependent factors at high concentrations. The main indication for prothrombin complex is in patients on anticoagulant therapy, such as warfarin, with significant bleeding or who require emergency surgery [46]. Conversely, single procoagulant factors are also available, most notably fibrinogen concentrate. As with desmopressin, these agents have the potential to increase the rates of thrombotic events. This has been shown clearly in clinical trials of recombinant factor VIIa in unselected patients [42].

Fibrinogen depletion, secondary to hemodilution and/or consumption, is thought to occur before deficiencies of other coagulation factors become apparent during hemorrhage [141]. Hypofibrinogenemia is a risk factor for hemorrhage in orthopedic surgery [54], cardiac surgery [55] and trauma [141]. In obstetric hemorrhage, a fibrinogen level below 2 g/l has a 100 per cent predictive value for progression from moderate to severe hemorrhage [56]. Fibrinogen concentrate is currently not licensed for acquired hypofibrinogenemia in the UK but is used widely in Europe. UK guidelines [57] recommend use of cryoprecipitate as the source of fibrinogen. However, off-label use has been reported, with small studies showing reduced red cell and fresh frozen plasma transfusion requirements [58]. Despite the physiological promise of procoagulant factors, high-quality data to guide safe and effective use are lacking. A recent systematic review [59] was unable to draw any conclusions owing to a paucity of data. However, the review identified 22 ongoing trials, so more definitive evidence regarding the safety, efficacy, and cost-effectiveness of procoagulant factors is likely to be available in the future.

## Recommendation 12: viscoelastic haemostatic assays help to identify the risk of bleeding or to minimise transfusion. (Grade of recommendation: 1C)

Viscoelastic hemostatic assays are increasingly being used in the management and prevention of severe bleeding. The two common assays are thromboelastography (TEG) and rotational thromboelastometry (ROTEM). The main advantage of these assays is the quick turnaround time, with an assessment of all stages of clot formation available in a few minutes. Standard laboratory tests such as prothrombin time can have a turnaround time of up to 77 min, which is not useful in a rapidly evolving situation [60].

Several guidelines recommend these assays only in patients undergoing cardiac and liver surgery, where robust cost-effectiveness data exist to support their use [62]. More recent guidance from the British Society of Haematology also suggests that TEG/ROTEM may have a role in the management of trauma and obstetric haemorrhage [61, 63, 142].

As previously stated in damage control management viscoelastic haemostatic assays are determinant in clinical decisions. They may potentially reduce the usage of blood products and preserve from unnecessary transfusions.

## Recommendation 13: maintain acute normovolemic haemodilution to help minimise bleeding and transfusion. (Grade of recommendation: 1C)

Acute normovolemic hemodilution (ANH) is a blood conservation technique consisting in removal of whole blood from a patient immediately after induction of anesthesia, with maintenance of normovolemia using fluid replacement with crystalloid and/or colloid. The amount of blood removed typically varies between 1 and 3 units (450 to 500 mL = one unit), although 3 to 4 units may be withdrawn safely in selected patients.

The main factors determining the efficacy of hemodilution are (I) Initial red cell mass where patients with greater red cell masses can provide more blood and this is mainly determined by the initial hematocrit and the blood volume.

(II) The magnitude of hemodilution. In fact, if lower hematocrits are achieved and tolerated by the patients after hemodilution, less red cell loss will be lost during surgical procedures. However, the more dilution is high the more profound are hemodynamic and physio-metabolic consequences. Red blood cells are important for hemostasis, and the risk of anemia has to be considered [143].

(III) Intraoperative blood loss.

(IV) Intraoperative management. Normovolemia should be effectively reestablished and managed after autologous blood removal. If this does not happen potential benefits in terms of reducing red cell losses may be vanished. In fact, in the absence of hemodilution, operative blood loss would occur as per preoperative hematocrit level.

The intraoperative lower hematocrit may request additional cardiovascular monitoring [144].

ANH can be used as the sole blood conservation technique, but for better results, it can be combined with other strategies to minimize or avoid transfusion.

Meta-analyses of RCTs indicate that ANH is effective in reducing the volume of allogeneic blood transfused and the number of patients transfused with allogeneic blood for major cardiac, orthopaedic, thoracic, or liver surgery. Additional meta- analyses of RCTs indicate that ANH combined with intraoperative red blood cell recovery compared with intraoperative red blood cell recovery alone is effective in reducing the volume of allogeneic blood transfused.

The most common contraindications for ANH are severe sepsis; acute respiratory insufficiency; acute renal failure and hemorrhagic shock secondary to trauma. Patients suffering from severe coronary arterial disease should only be assigned after careful assessments of the risks and benefits of this procedure [145]. Patients with surgical emergencies need to be well clinically evaluated before the indication of ANH.

## Recommendation 14: constantly monitoring of patient physiology is essential and, if the patient consents, transfuse according to well-founded clinical indications only.

### Special considerations are required for patients who decline transfusion (e.g. Jehovah’s Witness). (Grade of recommendation: 1C)

Although the aim would be to limit and minimise blood loss and hence the need for transfusions; inevitably transfusions may still be required. The commonest modality would be allogeneic red blood cell transfusions. Surgeons and anaesthetists must communicate frequently on the ongoing situation so that the team can stay on top of the blood loss and transfuse if needed. There is a need for ongoing monitoring of blood loss. This consists of visual assessment of the surgical field, including the extent of blood present, presence of microvascular bleeding, surgical sponges, clot size and shape, and volume in suction canister. Monitoring for perfusion of vital organs includes standard ASA monitoring. Additional monitoring may include echocardiography, renal monitoring (urine output), cerebral monitoring (*i.e.,* cerebral oximetry and near infrared spectroscopy [NIRS]), analysis of arterial blood gasses, and mixed venous oxygen saturation and haemoglobin/haematocrit levels. Monitoring for coagulopathy includes standard coagulation tests (*e.g.*, INR, activated partial thromboplastin time [aPTT], fibrinogen concentration), as well as platelet count. Additional monitoring for coagulopathy may include tests of platelet function, and viscoelastic assays (*e.g.,* TEG, ROTEM). An observational study examining platelet count during cardiopulmonary bypass to predict excessive blood loss reports a sensitivity value of 83% and a specificity value of 58%. An RCT reported not definitive findings for blood loss and transfusion requirements when TEG is compared with standard laboratory coagulation tests. An RCT reported equivocal findings with ROTEM *versus* no fibrinolysis monitoring for RBC, FFP, and platelet transfusion requirements. TEG and ROTEM-guided algorithms are shown to be effective in reducing blood transfusion requirements. For ROTEM, a sensitivity finding for blood loss was reported to be 13%, specificity values ranged from 52 to 80%, and a positive predictive value of 45%. Nonrandomized correlational studies reported significant correlations (*P* < 0.01) with standard coagulation tests for fibrinogen level and platelet count, whereas correlations between ROTEM and prothrombin time (PT) and aPTT measures were not statistically significant.

Nonrandomized comparative studies report higher risk of infection after RBC transfusion and case reports indicate that adverse outcomes including transfusion-related acute lung injury and delayed haemolytic transfusion reaction may occur after transfusion.

**Recommendation 15: physicians must respect local laws and accept that competent patients have the right to decline blood transfusion. Physicians maintain the right and duty to decide whether they can perform a specific procedure without blood transfusion. If they cannot perform a procedure without blood transfusion, they should consider other therapeutic options.**

## Specific consideration must be reserved to unemancipated minors and patients unable to understand and want. (Grade of recommendation: 1C)

There are patients who may decline blood transfusions for various reasons, most commonly due to religious beliefs. The most prominent group of patients in this category are the Jehovah’s Witness (JWs) (Table 1). Some national well known health services and associations developed recommendations for surgeons caring for patients declining blood transfusion. In general surgeons highly value and respect patient autonomy and simultaneously desire to provide high-quality and effective treatment. On the other hand, exists necessity to contribute to prevent the death of a patient. This may be even more difficult to achieve in those patients whose death might be prevented by transfusing blood. To provide optimal care for patients who are JWs, surgeons should aim to respect and accommodate each patient’s values and target the best outcome possible given the patient’s desires and his/her clinical condition. As this topic presents many different and articulated arguments, it is not possible to provide blanket guidelines or standards for the care of JWs and all those patients who may decline blood transfusion. The attempt to delineate precise recommendations about when and what to use blood product, especially in emergency setting, may bring serious unintended consequences. Each person declining blood transfusions, whether for religious or personal reasons, is unique, independent and possesses personal wishes, values, and beliefs about what is acceptable therapy. It is crucial that surgeons and patients accurately discuss what they may accept or not in term of blood product and also the circumstances and the criticalities that may present during emergency management of acute surgical or traumatic illness. It should be noted that many patients who decline blood transfusion (i.e., whole blood, red blood cells, white cells, platelets, plasma) for religious reasons will permit the use of cell salvage and many other blood conservation modalities. (See table below.) Moreover, many will accept derivatives of primary blood components (“blood fractions”) (e.g., clotting factor concentrates, albumin, immunoglobulins). The community help is fundamental. In fact, many JW patients have already clarified their own beliefs regarding transfusions and usually have some documents outlying very clearly their preferences. Those patients declining blood transfusions very often present for treatment with relatives or other representatives of their faith helping them during the hospital admission and ensuring that their treatment instructions are respected. On the other hand, clinicians should conduct an informed consent discussion by offering information in language the patient will understand of the various therapeutic options and the risks and benefits of each option. Clinicians should avoid generalization and consult with individual patients as to what each one’s religious conscience dictates. Some patients might accept products containing derivatives of either primary cellular components or plasma, or surgical or extracorporeal blood management methods, whereas others may refuse them. Such a discussion must be done without coercion. The right to privacy must be respected and the patient must have if needed the time to discuss alone with the doctors about their beliefs and requests. Physicians must respect each patient’s will, but also explain the potential consequences of declining blood transfusion. During treatment, the patient is free to change his/her mind. In fact, some may very clearly state their preference to decline allogeneic blood transfusion in nonemergent and non-life-threatening situations but may change their mind if their life is a risk. All patients must be warranted the right to change their mind. It is the surgeon’s responsibility to clarify, without any antagonism the patient’s preference to decline transfusion is stable and would not change if there was a risk to life. In the emergency setting, whenever the patients may not have the capacity to express their will, the decisional process about if to proceed with transfusion or not may potentially delay the treatment and lead to the patient’s death. Treatment delays can be avoided by reviewing an incapable patient’s advance medical directive (Durable Power of Attorney), recent record of hospitalization, or speaking with the patient’s appointed substitute decision-maker. If there is no clear indication of the patient’s treatment instructions or relevant wishes, surgeons are on solid ground in treating the patient using all available modalities under the standard of presumed consent. When a patient has an advance directive clearly refusing allogeneic blood transfusion, that directive must be followed. In the case of unemancipated patients and or children the management must in general warrant the right of the patient to be cured. Legal tutors have limited right to manage certain life-threatening behaviours and it must be case-by case referred to the local country and state laws.

## Patient care when blood transfusion is not an option

Medical care without allogeneic blood transfusion (bloodless medicine and surgery) must be considered in a number of clinical settings: [1] when patients decline allogeneic blood transfusion for religious (e.g., JWs, see Table 2) or personal reasons; [2] when blood may be in short supply (e.g., trauma, mass casualty events, and critical inventory shortages continue to occur in Canada, the United States and some European nations since the pandemic); [3] when safe blood (i.e., screened and tested) is not available (one out of four low-income countries do not test all donated blood for infectious diseases and 54% of countries do not have blood surveillance systems, according to the World Health Organization); [4] when compatible blood transfusion may be unobtainable (e.g., for patients with multiple allo-antibodies). In each of the above settings, clinicians need to adopt of philosophy of rigorous blood conservation. Moreover, a philosophy of patient blood management that incorporates bloodless medicine principles is appropriate for all patients. Many reports of patients treated without transfusion for a variety of conditions show that preempting, minimizing, or avoiding of allogeneic blood is safe and effective [146]. Bloodless medicine is still in the process of development and is not without limitations. Strategies for managing acute, severe anemia continue to evolve [147].

**Table 2:** Position of Jehovah’s Witnesses on Medical Therapy (* Cryoprecipitate using 0.9% Sodium Chloride Injection (USP) diluent)

A: Acceptable Treatment
Most surgical and anaesthesiological blood conservation measures (e.g., haemostatic surgical instruments, controlled hypotension/hypotensive haemostasis, regional anaesthesia, minimally invasive surgery, endovascular therapy, intraoperative positioning, maintenance of normothermia, meticulous haemostasis and surgical technique) • Most diagnostic and therapeutic procedures (e.g., phlebotomy for laboratory testing, angiographic embolization) • Pharmacologic agents that do not contain blood components or fractions such as: • Drugs to enhance haemostasis (e.g., tranexamic acid, epsilon-aminocaproic acid, aprotinin, desmopressin, conjugated oestrogens) • Hematopoietic growth factors and hematinics (e.g., albumin-free erythropoietin, iron) • Recombinant products (e.g., albumin-free coagulation factors) • Topical haemostatic agents (e.g., collagen, gelatin-based haemostats, oxidized cellulose) • Synthetic oxygen therapeutics (e.g., perfluorochemicals) • Nonblood volume expanders (e.g., saline, lactated Ringer’s, Hartmann’s solution, starch solutions)
B: Personal Decision (Acceptable to Some, Declined by Others)
• Blood cell salvage (intraoperative or postoperative autotransfusion) (patients might request that continuity with their vascular system be maintained) • Acute normovolaemic haemodilution (patients might request that continuity with their vascular system be maintained) • Intraoperative autologous blood component sequestration (including intraoperative plateletpheresis, preparation of fibrin gel, platelet gel, platelet-rich plasma) (patients might request that continuity with their vascular system be maintained) • Cardiopulmonary bypass (circuit not primed with allogeneic blood) • Apheresis (circuit not primed with allogeneic blood) • Haemodialysis (circuit not primed with allogeneic blood) Plasma-derived fractions (e.g., fibrinogen, prothrombin complex concentrates, immune globulins, antivenins, vaccines, albumin, cryoprecipitate*) • Haemostatic products containing blood fractions (e.g., recombinant factor VIIa, coagulation factor concentrates, prothrombin complex concentrate, fibrin glue/sealant, haemostatic bandages containing plasma fractions, thrombin sealants) • Products containing plasma-derived blood fractions such as human serum albumin (e.g., some formulations of erythropoietin, streptokinase, G-CSF, vaccines, recombinant clotting factors, nuclear imaging products) • Products containing a blood cell-derived fraction, whether from a human or animal source (e.g., hemin, oxygen therapeutics) • Epidural blood patch • Blood cell scintigraphy (e.g., radionuclide tagging for localization of bleeding) • Peripheral blood stem cell transplantation (autologous or allogeneic) • Transplants (organ, marrow, bone)
C: Unacceptable Treatment
• Transfusion of allogeneic whole blood, red blood cells, white cells, platelets, or plasma • Preoperative autologous blood donation (PAD)

## Conclusion

The potential for major intraoperative blood loss remains a key concern for surgeons and anesthetists. The management of anemia, optimization of coagulation status, and rigorous minimization of any blood loss during surgery involves a multimodal and multidisciplinary approach, which includes preoperative, intraoperative, and postoperative strategies. Strategies to mitigate this, such as identification and management of high-risk patients, can be implemented early in the preoperative phase. During surgery, meticulous surgical techniques and local hemostasis are fundamental measures in the control of bleeding. Cell salvage is a valuable adjunct. Tranexamic acid reduces blood loss, but the optimal route, dose and timing of administration remain unclear. Additional anesthetic techniques, such as regional anesthesia, maintenance of normothermia, and controlled hypotensive anesthesia can also help to reduce blood loss. Other key considerations include avoidance of hypothermia, acidosis and excessive hemodilution along with early identification of coagulopathy using viscoelastic hemostatic assays. The optimal use of hemostatic therapies such as desmopressin and procoagulant factors is unclear at present, but rep- resents an important area of ongoing research. Close collaboration between anesthetists, surgeons, hematologists, and laboratory personnel is vital. For patients who decline blood transfusions, the managing specialists needs to handle that situation with care and empathy. The specialist must also have a clear understanding of his or her country’s state and laws. This is always a difficult issue that can benefit from greater national and legal clarity to be able to balance between respecting autonomy against non-maleficence. Ultimately, the optimal use of blood transfusion is dependent on a careful collaboration and optimization of the clinical pathway, from prehospital until rehabilitation and reintegration to community, by a multidisciplinary team of practitioners.

## Data Availability

Not applicable.

## Abbreviations

PBM: Patient blood management
DOAC: Direct oral anticoagulant
WHO: World health organization
RCT: Randomized controlled trials
PCCs: Prothrombin complex concentrates
FFP: Fresh frozen plasma
INR: International normalized ratio
TXA: Tranexamic acid
VET: Visco-elastic-testing
ROTEM: Rotational thromboelastometry
TEG: Thromboelastography
MTP: Massive transfusion protocols
DCS: Damage control surgery
PEEP: Positive end-expiratory pressure
ANH: Acute normovolemic hemodilution
aPTT: Partial thromboplastin time
JWs: Jehovah’s witness
